# Rapid-cycle systems modeling to support evidence-informed decision-making during system-wide implementation

**DOI:** 10.1186/s43058-021-00218-6

**Published:** 2021-10-09

**Authors:** R. Christopher Sheldrick, Gracelyn Cruden, Ana J. Schaefer, Thomas I. Mackie

**Affiliations:** 1 Department of Health Law, Policy and Management, School of Public Health, Boston University, One Silber Way, Boston, MA USA; 2 Oregon Social Learning Center, 10 Shelton McMurphey Blvd, Eugene, OR USA; 3 SUNY Downstate Health Sciences University, 450 Clarkson Ave, Brooklyn, NY USA

**Keywords:** Computer simulation, Epistemology, Implementation science, Evidence-based practice, Psychological trauma, Screening

## Abstract

**Background:**

To “model and simulate change” is an accepted strategy to support implementation at scale. Much like a power analysis can inform decisions about study design, simulation models offer an *analytic strategy* to synthesize evidence that informs decisions regarding implementation of evidence-based interventions. However, simulation modeling is under-utilized in implementation science. To realize the potential of simulation modeling as an *implementation strategy*, additional methods are required to assist stakeholders to use models to examine underlying assumptions, consider alternative strategies, and anticipate downstream consequences of implementation. To this end, we propose Rapid-cycle Systems Modeling (RCSM)—a form of group modeling designed to promote engagement with evidence to support implementation. To demonstrate its utility, we provide an illustrative case study with mid-level administrators developing system-wide interventions that aim to identify and treat trauma among children entering foster care.

**Methods:**

RCSM is an iterative method that includes three steps per cycle: (1) identify and prioritize stakeholder questions, (2) develop or refine a simulation model, and (3) engage in dialogue regarding model relevance, insights, and utility for implementation. For the case study, 31 key informants were engaged in step 1, a prior simulation model was adapted for step 2, and six member-checking group interviews (*n* = 16) were conducted for step 3.

**Results:**

Step 1 engaged qualitative methods to identify and prioritize stakeholder questions, specifically identifying a set of inter-related decisions to promote implementing trauma-informed screening. In step 2, the research team created a presentation to communicate key findings from the simulation model that addressed decisions about programmatic reach, optimal screening thresholds to balance demand for treatment with supply, capacity to start-up and sustain screening, and availability of downstream capacity to provide treatment for those with indicated need. In step 3, member-checking group interviews with stakeholders documented the relevance of the model results to implementation decisions, insight regarding opportunities to improve system performance, and potential to inform conversations regarding anticipated implications of implementation choices.

**Conclusions:**

By embedding simulation modeling in a process of stakeholder engagement, RCSM offers guidance to realize the potential of modeling not only as an analytic strategy, but also as an implementation strategy.

Contributions to the literature

- Simulation modeling is accepted as an *analytic strategy* in implementation science.
- To realize the potential of simulation modeling as an *implementation strategy*, we propose Rapid-cycle Systems Modeling (RCSM).
- RCSM helps stakeholders leverage evidence to inform decisions by participating in dialogue about and simulation modeling of the implementation process.
- RCSM can help decision-makers leverage existing evidence to anticipate downstream unintended consequences, identify opportunities to improve system performance, and plan appropriate evaluation strategies.

## Background

The success of both system-wide innovations and evidence-based practices depends on implementation strategies that effectively promote adoption, sustainment, and dissemination at scale [1–3]. As articulated in the Expert Recommendations for Implementing Change (ERIC), one promising strategy is to “model and simulate change” (p. 6). Given the rapid growth of simulation modeling in the health sciences [4–6], there are increasing calls for greater use in implementation science to promote evidence-informed decision-making [7, 8]. However, at its core, simulation modeling is a quantitative method widely used as an *analytic strategy*. To facilitate its use as an *implementation strategy*, the current paper presents a method referred to as Rapid-cycle Systems Modeling (RCSM)—a three-step, cyclical method designed to realize the benefits of simulation modeling for implementation science. Specifically, we describe the evidence and theory underlying the two major components of RCSM: (1) the simulation model, itself, and (2) the process of stakeholder engagement necessary to realize its full potential as an implementation strategy. We then present a case study to demonstrate the utility of RCSM for implementation.

### Simulation modeling to promote evidence-informed decision-making

Despite rapid growth in some fields, simulation modeling remains under-utilized, especially in implementation science [4–6]. One clear barrier is the lack of familiarity with simulation modeling among core constituencies. As one paper noted [9], “clinicians and scientists working in public health are somewhat befuddled by this methodology that at times appears to be radically different from analytic methods, such as statistical modeling, to which the researchers are accustomed,” (p. 123S). Simulation modeling represents a way of thinking that differs from the inductive logic underlying most empirical methods. Rather than beginning with observed data and then generating inferences, simulation modeling typically involves “reasoning to the best explanation,” a form of logic known as abduction that was first described by the pragmatic philosopher, Charles Sanders Peirce, and is common throughout all branches of science [10, 11].

Notably, one form of simulation modeling is already widely accepted by health researchers: power analysis. By definition, research studies are intended to investigate areas of scientific uncertainty, yet this uncertainty creates challenges for developing a priori study designs. Prior to clinical trials, for example, researchers gather evidence to inform assumptions regarding expected treatment effect, consider their risk preferences regarding type 1 and type 2 errors, and apply statistical expertise to estimate an optimal sample size. Often, researchers consider a range of plausible effect sizes that are consistent with available evidence and risk preferences (e.g., 90 or 80% power). Ultimately, researchers settle on the power calculation deemed most appropriate and use it to justify and inform decisions regarding sample size.

In similar ways, simulation models of many kinds can support evidence-informed decision-making for implementation of system-wide innovations. Indeed, we argue that implementation scientists should not expect a system-wide innovation to realize a net benefit within a given context without first ensuring that the assumptions of their implementation design are consistent with prior evidence and that potential risks are acceptable. Such judgments can be meaningfully informed by simulation modeling. Furthermore, simulation modeling can inform the implementation process by broadening consideration of candidate implementation strategies (e.g., by linking to fields such as operations research), deepening the search for implementation barriers and facilitators (e.g., by considering dynamic complexity and policy resistance), and facilitating outcome evaluations (e.g., by identifying full cascades of potential effects—both intended and unintended).

### Simulation modeling as an analytic strategy

Simulation modeling offers a flexible approach to synthesizing research evidence and applying it to a range of decisions necessary for system-wide innovations. To cite one example, a recent systematic literature review was conducted to inform a state-level effort to implement screening for adverse childhood experiences (ACEs) in pediatric settings [12]. Whereas meta-analysis synthesizes evidence across multiple studies to estimate a single parameter (e.g., prevalence or screening sensitivity), simulation modeling offers the flexibility to synthesize disparate forms of evidence while considering distal outcomes. In this case, the authors analyzed potential implications of screening implementation by applying available research evidence to a simple simulation model of the clinical pathway from detection to intervention. Results demonstrated that extant evidence is consistent with a wide range of scenarios in which implementation of ACEs screening induces anything from modest decreases in demand for services to very large increases. While available evidence was found to be insufficient to support precise predictions, results highlighted the importance of monitoring demand and attending to workforce capacity, as well as the potential of leveraging existing datasets to address evidence gaps in operations outcomes following screening implementation.

The process of simulating possible implementation scenarios holds an additional benefit: simulation often promotes insight. While seldom defined or operationalized, modelers often use the term “insight” to refer to lessons learned regarding the causal determinants of a given problem [13–15], the net value of and/or tradeoffs inherent in potential solutions [15–17], unrecognized evidence gaps [15], unexpected results [16], or sensitivity to the metrics used to measure outcomes [16]. Notably, in none of these instances does “insight” refer to a precise estimate or a statement of truth, as is the typical goal of inductive and deductive logic, respectively. Instead, all provide examples of learnings that support abductive logic, often through careful examination of underlying assumptions.

Concretely, the act of simply writing out all the parameters required to specify even a simple simulation model begins to make explicit the assumptions that underlie expectations. For example, simulating the number of patients who will require treatment after implementing a screening program minimally requires estimates of underlying prevalence, screening tool accuracy (e.g., sensitivity and specificity), and the probability that referrals will be offered and completed. Identifying underlying assumptions can thus reveal important evidence gaps, highlighting the minimal amount of evidence required to understand a system. In the words of one famous modeler [18], “uncertainty seeps in through every pore” (p. 828), even for seemingly simple problems. In particular, system-wide innovations generally enjoy an evidence base that is less robust than for clinical interventions, which are more often subject to randomized trials and are more easily standardized [19].

Moreover, consideration of underlying assumptions can facilitate understandings of alternative strategies that target different points in a larger system. For example, a simulation model designed to understand clinical decision-making for behavioral interventions suggested multiple strategies for improving early detection including not only screening, but also audit-and-feedback to improve error rates and integrated behavioral health services to facilitate referrals and reduce the perceived cost of false positive results [20].

Equally important, simulation models can reveal implicit assumptions that are inconsistent or contradictory [21]. For example, one might assume that as long as capacity to provide treatment exceeds demand, waitlists should not present a problem. However, even the simple simulation model described above was capable of demonstrating complex interactions between supply and demand, including how waitlists can emerge despite significant capacity [22]. For example, a missed appointment can expend an hour of a treatment provider’s time (if they cannot immediately schedule another patient) while simultaneously adding to the waitlist (assuming the patient reschedules). Thus, it may not be enough to offer more treatment hours: mechanisms to manage missed appointments might also be considered during implementation planning. Waitlists are a classic operational research problem; as Monks [22] argues, simulation modeling forms the foundation of operational research, which can address logistical problems and optimize healthcare delivery [22].

At a deeper level, simulation models can help address foundational assumptions of the statistical models employed when planning and evaluating system-wide interventions. As Raghavan [23] argues, prevailing conceptual models for system-wide interventions are typically multidimensional and complex, often positing mutual interactions between variables at different socioecological levels (e.g., sociopolitical, regulatory and purchasing agency, organizational, interpersonal; 23). Many of these relationships involve reciprocal causation—i.e., when two variables are each a cause of the other. Whereas most inferential statistics based on the general linear model fail to address reciprocal causation—in fact, they assume it does not exist [24–26]—simulation models address reciprocal causation through the concept of feedback loops, in which changes in one variable cause consistent changes in associated variables (reinforcing loops) or mitigate such changes (balancing loops [27];). System dynamics—a field of simulation modeling with a strong focus on feedback loops—suggests that we, as implementation scientists, ignore reciprocal causation at our peril. Dynamically complex systems marked by reciprocal causation, feedback loops, time delays, and non-linear effects often exhibit policy resistance—that is, situations where seemingly obvious solutions do not work as well as intended, or even make the problem worse [28]. Examples of systems-level resistance to innovations are common, such as the historic trend toward larger, more severe forest fires in response to fire suppression efforts or the rapid evolution of resistant bacteria in the face of widespread use of antibiotics. As Sterman [28] points out, the consequences of interventions in dynamically complex systems are seldom evident to those who first implemented them. Simulation modeling offers a quantitative method to uncover and address the underlying assumptions of system-wide interventions, thus facilitating the identification of potential implementation barriers (e.g., feedback loops driving adverse outcomes) early in the planning process. In this way, simulation modeling can refine “mental models”—human’s internal understandings of an external system—which are often both limited and enduring [29]. For example, the ACEs screening model [12] demonstrates the potential for treatment capacity to be influenced through balancing and/or reinforcing feedback loops involving waitlists and staff burnout—both of which introduce the potential for dynamic complexity and policy resistance. Simulation modeling thus offers an opportunity for careful reflection about the complex dynamics in which many interventions function as elements of the systems they are designed to influence [30].

### Simulation modeling as an implementation strategy

As an analytic strategy, simulation modeling can help synthesize a range of available evidence applicable to a given implementation challenge while making underlying assumptions explicit. But analysis is only half the battle. If assumptions appear solely in the “fine print” of a model’s computer code, they are unlikely to be understood, interrogated, or challenged by other stakeholders. Engagement is needed to realize simulation modeling’s full value. Here, we argue that to be an effective implementation strategy, simulation modeling is best implemented in the context of cultural exchange—i.e., an in-depth process of negotiation and compromise between stakeholders and model developers [31]. In turn, stakeholder participation can improve the analytic value of the models themselves. Concretely, making assumptions explicit through simulation models allows for their refinement and critique through dialogue between researchers and stakeholders, including clarification of their frequently divergent assumptions, sources of evidence, and priorities.

The importance of engagement in the modeling process has empirical support. Decision-makers have endorsed the “co-production” of simulation models, citing the insights gained, the desirability of simulating proposed interventions effects prior to implementation, and the identification of evidence gaps [32]. The process of negotiation and compromise while co-producing models has been found to influence decision-makers’ attitudes, subjective norms, and intentions [33], which help achieve alignment and promote community action [34, 35]. These findings are consistent with observations in management science from over 50 years ago [22, 36], as well as recent research on cultural exchange theory demonstrating that dialogue, negotiation, and compromise between scientists and implementers can directly contribute to implementation success [31].

Consistent with contemporary epistemology, this perspective on modeling suggests that application of the scientific method is not sufficient to prevent bias or error and that findings are imbued with theory and values that are influenced by social context [37, 38]. As a remedy, theories of situated knowledge advocate for “critical interaction among the members of the scientific community [and] among members of different communities” [39] as the best way to discern scientific assumptions and address their potential consequences. Consistent with this focus, system dynamics is explicitly intended to help scientists uncover hidden assumptions and biases [40] based on recognition of the limits of traditional research methodologies as well as the observation that “we are not only failing to solve the persistent problems we face but are in fact causing them.” ( [28] , p.501) Recognizing the benefit of uncovering hidden assumptions and biases in our scientific understandings holds profound implications, shifting our translational efforts from uptake of research evidence alone to promoting the bidirectional exchange of evidence, expertise, and values [41].

To facilitate cultural exchange of this kind, RCSM emphasizes dialogue among all relevant stakeholders (e.g., decision-makers, model developers, researchers). Dialogue theory describes different forms of relevant interactions [42]. For example, shared inquiry is initially necessary to gain a mutual understanding of available evidence and relevant priorities. As stakeholders develop opinions about possible implementation strategies and their implications, critical discussions can ensue about their relative merits, using the simulation model as an interrogation guide. Finally, when the time and cost of further critical discussions outweigh their benefits, a simulation model can guide deliberations about how implementation should proceed and be monitored and evaluated. The effectiveness of the simulation model can thus be assessed by its relevance to implementation decisions, the insight it elicits, and its utility for further planning.

However, there is not enough concrete guidance on how to promote engagement with simulation models to support implementation efforts. To fill this gap, RCSM uses an approach similar to group model building (GMB), which is a process of engagement with system dynamic models and systems thinking that is well-suited to facilitate use of simulation modeling in implementation science [43]. Several GMB principles are conceptualized as core attributes of RCSM. Both are “participatory method[s] for involving communities in the process of understanding and changing systems…,” ( [44] , p. 1) both emphasize scientific uncertainty and the questioning of assumptions, and both focus on collaboration between stakeholders and simulation modelers across multiple stages, from problem formulation to generating consensus regarding strategies for intervention [45]. However, use of GMB in implementation science has been limited. Building on GMB, RCSM targets the needs of implementers by focusing on rapid cycles that can fit within short policy windows. Moreover, RCSM is not limited to system dynamics, but is open to any form of simulation modeling that can usefully address decision-makers’ questions with transparency. For example, whereas the screening example described above involved a Monte-Carlo simulation, other types of models are also possible, including microsimulation, agent-based modeling, Markov modeling, and discrete-event simulation. At its core, RCSM is a pragmatic approach that is designed to be responsive to decision-makers’ needs.

For readers interested in greater detail regarding modeling approaches and their applications, we recommend reviews focusing on management [46] and healthcare [47, 48]. For those interested in learning to build simulation models, we found Baker’s description of how to develop basic optimization models using Excel [49] to be invaluable, as is Sterman’s detailed text on system dynamics [50].

### Case Study: Rapid-cycle Systems Modeling (RCSM) of trauma-informed screening

To explain RCSM’s rationale and demonstrate its use, we report an illustrative example of an initial cycle of RCSM conducted with state-level decision-makers seeking to promote trauma-informed screening programs for children and adolescents (“youth”) in foster care. In response to federal legislation, U.S. states have been working to implement trauma-informed screening and evaluation for children in foster care over the past decade [51, 52]. This case example builds on prior studies investigating the role of mid-level administrators’ use of research evidence while enacting statewide innovations for youth in foster care [3, 52].

## Methods

RCSM involves a process of iterative, stakeholder-engaged design to test the assumptions that underlie system-wide innovation and implementation. Consistent with traditions in evidence-based medicine that derive from decision analysis, RCSM recognizes the need for the best available scientific evidence, the expertise to address scientific uncertainty in the application of that evidence, and stakeholder values to define model scope and purpose and to weigh tradeoffs between competing outcomes [53]. To accomplish these goals, each cycle of simulation modeling in RCSM involves three steps: (1) identify and prioritize stakeholder questions, (2) develop or refine a simulation model, and (3) engage in dialogue regarding model relevance, insights, and utility for implementation. This final step can inform prioritization of stakeholder questions for future cycles of RCSM.

Below, we describe each of the three steps in the RCSM cycle. Table 1 provides an overview of how RCSM is operationalized in this case study.

**Table 1:** Rapid-cycle Systems Modeling: illustrative case study

Activity	Goal	Potential methods	Products	Illustrative case study
**RCSM Step 1: Identify the relevant stakeholder questions**	Identify relevant stakeholders and the questions and priorities involved in the decisions they confront.	• Interviews • Focus groups • Surveys • Observations	Sampling framework and documentation of stakeholder questions and priorities	• *Goal*: To identify questions and decisions confronted by mid-level managers when developing universal trauma screening and/or assessment protocols for youth entering foster care. • *Method*: Sampling framework sought key informants who were engaged in decision-making processes regarding the provision of trauma-informed services for children in foster care. Approximately hour-long semi-structured interviews were conducted by telephone with 31 key informants across 12 states. Key informants were mid-level administrators from Medicaid, child welfare, and mental health agencies with roles developing policy for the provision of trauma-informed services for children in foster care. The interview guide was based on a decision sampling framework, with questions grounded in core domains of decision analysis. Respondents were sampled until no new themes in the core domains emerged (i.e., thematic saturation). Trained qualitative researchers (TM, AS, BF, ER) conducted interviews and analysis at their respective research institute. Research team engaged a modified framework analysis in DeDoose^TM^, consisting of seven steps to identify and index the specific decisions relevant to the implementation of trauma-informed screening. The research protocol for the illustrative case study was reviewed and approved by the Institutional Review Board at [withheld to preserve anonymity]. Additional details of the methodological approach are previously published [citation with to preserve anonymity]. • *Product*: Decision set of five decision points (see Results).
**RCSM Step 2: Develop Simulation Model**	Develop and/or refine a simple simulation model to address the questions identified in Step 1.	• Monte-Carlo model • Discrete-event model • System dynamics • Agent-based model	• Simulation model • Evidence synthesis	• *Goal*: Refine a simulation model and conduct virtual experiments that address questions relevant to statewide implementation of trauma-informed screening • *Method*: Adapted a Monte-Carlo model of a typical screening process (hereafter, the “baseline model”; see Fig. 1) from previous research [12]. Virtual experiments focused on the sensitivity of the overall process for moving children with trauma to treatment, the false positive rate, influence of screening on demand for services, workforce capacity to provide treatment, and the potential for waitlists if demand exceeds supply. To address stakeholders’ questions about merits of altering screening thresholds, sensitivity analyses focused on an increase in screening thresholds, which increases specificity but lowers sensitivity. • *Product*: Simulation model synthesized evidence from systematic reviews; a slide deck and presentation detailed the Monte-Carlo model, analytic results, and results relevant to each question from step 1.
**RCSM Step 3. Stakeholder engagement with iterated simulation model**	• Assess relevance of model to stakeholder decisions • Seek insight into question • Discuss utility for informed decision-making in support of implementation	• Identify relevant stakeholders for iterative model development. • Validation of qualitative data to search for disconfirming evidence, probe underlying assumptions • Group dialog in service of inquiry into evidence and its application, critical discussion of competing hypotheses, and deliberation regarding best course of action	• Identification of alternative strategies • Identification of potential barriers & mitigation plans • Articulate hypotheses regarding key causal mechanisms	• *Goal*: To assess model relevance, seek insight regarding systemic factors likely to drive success, and discuss model utility to support implementation • *Method*: To provide input, we sampled both intermediaries and a subset of key informants initially interviewed in Step 1. Trained qualitative researchers [TM, AS] convened four member-checking group interviews through an online platform with key informants (*n* = 8) of the 31 key informants engaged in Step 1 semi-structured interviews and two additional group interviews with “intermediaries” (*n* = 8) who developed, evaluated and/or provided technical assistance for mental health screening and trauma-specific interventions. The study team presented a standardized slide deck. Respondents were provided findings and asked questions after each section. Following the presentation of the simulation model, respondents were asked “Does this model seem applicable to your delivery system? If so, how?”, “How, if at all, would you want to change the model to accommodate your delivery system?” and “What would be the strengths or limitations of this model when applied to your delivery system?” Each member-checking group interview transcript was analyzed following completion. We used an immersion-crystallization approach in which two study team members listened to and read each group interview to identify important concepts and engaged open coding and memos to identify themes and disconfirming evidence. Additional details of the methodological approach are previously published [citation with to preserve anonymity]. • *Product*. Summary of utility and potential modifications to customize to decision-maker needs.

### RCSM Step 1: Identify stakeholders’ questions

Given RCSM’s focus on the needs of decision-makers, an understanding of the organizational and interpersonal processes in place for decision-making is critical to determination of the appropriate sampling framework [41]. The first task is to identify the individuals who inform or make the decisions pertinent to the policy or programmatic domain of interest. Sample selection criteria are consistent with key informant interviews, in which individuals are selected because they are deemed most knowledgeable about the phenomenon of interest, in this case decision-making in the domain of interest [54]. Consideration should also be given to the value of triangulating perspectives on a particular policy domain and attempting to secure a sample sufficient for qualitative standards of sample size (e.g., thematic saturation [55];).

To identify the questions of relevance to stakeholders, multiple qualitative approaches in the postpositivist tradition could be engaged, including interviews, surveys, or observation, so long as they provide sufficient detail to guide model development, including defining the models’ purpose, scope, structure, and opportunities for application. For example, our team relied on “decision sampling” to analyze the decisions confronted by mid-level policymakers. Based explicitly on decision analysis [53], the interview guide included questions on (1) decision points, (2) choices considered, (3) evidence and expertise regarding chance and outcomes, (4) outcomes prioritized, (5) expressed values, (6) tradeoffs considered in making the final decision, and (7) aspects of the decision-making process [41], itself. As detailed in a recent publication [41], decision sampling facilitated documentation of stakeholder questions and priorities, specifically through identification of specific questions relevant to actual decisions confronted by policymakers, which helped to articulate model purpose and scope.

### RCSM Step 2: Develop the simulation model

The goal of step 2 is to develop a simple simulation model that addresses stakeholder questions and to conduct preliminary “virtual experiments” relevant to implementation. In RCSM, model selection is pragmatic, considering the cost of model development alongside potential benefits. Clearly, the potential of modeling as an analytic tool increases with advances in the field, such as incorporation of Bayesian priors during sensitivity testing and use of simulation models to support causal inference [56, 57]. But just as a simple online calculator can inform the initial stages of a power analysis, simple simulation models can help inform implementation planning. Because they are more tractable and transparent than complex models, simple models may be more easily understood and therefore more likely to influence how researchers and decision-makers conceptualize problems [50, 58]. Simple models can also be developed more rapidly, thereby taking advantage of available policy windows (not to mention requests for proposals). Additionally, rapid results facilitate iterations of RCSM, which can include group decisions about the value of further model building (versus competing priorities, like further data collection) as well as adjustments to model scope and priorities. Although expert modelers might be engaged to develop more complex simulations, many researchers are capable of developing and applying simple simulation models early in the planning process, thereby helping to reveal the assumptions necessary for successful implementation of an innovation in a given system. Products of this step can include the simulation model itself, but also a report detailing how the model synthesizes available evidence with respect to stakeholders’ questions (e.g., see evidence synthesis on ACEs screening cited above [12];).

### RCSM Step 3: Stakeholder engagement with iterated simulation model

After discerning stakeholders’ questions (step 1) and attempting to formulate a helpful response (step 2), an important third step is to reconcile the two through dialogue. A primary purpose of RCSM is to examine implicit assumptions, including about what messages are heard or what models might be helpful. Accordingly, Step 3 prioritizes engagement between the stakeholders, the research team, and the model itself. Concretely, this step aims to ensure (1) relevance of the model to stakeholder needs, (2) potential for analytic insight into system-level factors that may influence implementation, and (3) utility to facilitate evidence-informed decision-making at a group level to advance implementation.

In this step, relevant stakeholders might include a wide array of individuals who could help to assess the relevance, accuracy, and potential application to the policy or programmatic innovation of interest. Stakeholders engaged in this step may be more broadly defined than in Step 1 so as to facilitate the assessment and interpretation of the model developed. Potential stakeholders to be engaged could align as broadly with the 7Ps framework for stakeholder engagement, including patients and the public, providers, purchasers, payers, policymakers, product makers, and principal investigators [59].

Consistent with tenets of data validation in qualitative research [60], this step prioritizes a search for disconfirming perspectives on simulation findings to help interrogate assumptions [61]. Products of this step often include “insight,” such as identification of potential barriers, mitigation plans, and alternative strategies consistent with implementation science frameworks emphasizing the role of inner and outer contexts. Engagement can also help stakeholders to articulate hypotheses regarding key causal mechanisms of intervention and implementation strategies, including their interaction and dependence on context. For example, the evidence synthesis described above articulated how the impact of ACE screening may depend on variables interacting at multiple levels, including screening accuracy, workforce capacity, and trust between patients and their providers [12]. This model could facilitate extension of hypothesized mechanisms to include outer context, for example by modeling the potential impact of state-level policy decisions on workforce capacity.

## Results

### RCSM Step 1: Identify stakeholders’ questions

Interviews documented a set of discrete and inter-related decisions required to promote implementation of trauma-informed screening. As reported elsewhere [41], implementation decisions with respect to trauma-informed screening were classified into five domains:

1. *Reach of the screening program*, including which children to screen and at what ages.
2. *Content of the screening tool*, including which screening tool to use, and whether it should directly assess traumatic life events, the sequelae of traumatic life events (e.g., symptoms), or both.
3. *Threshold or “cut-score” for referral*, including whether to adopt a threshold higher than is recommended in the research literature to avoid spikes in demand.
4. *Resources for screening start-up and sustainment*, such as whether sufficient resources are available in local systems to successfully implement screening.
5. *Downstream system capacity to respond*, such as whether sufficient resources are available in local systems to address downstream needs identified through screening, for example, need for intervention.

### RCSM Step 2: Develop simulation model

Our team selected a Monte-Carlo model for two primary reasons: (1) development time and cost was low because a preliminary model had already been created and many relevant parameters could be estimated based on extant data, and (2) relevance to stakeholder questions was likely given that proof-of-principle had been demonstrated for similar screening interventions [12], for example, by demonstrating the tradeoffs inherent in choice of screening cut-scores [62, 63]. To facilitate use, we built our Monte-Carlo model using widely available software (Microsoft Excel) that has been used to facilitate dissemination of optimization modeling [49].

**Fig. 1.**
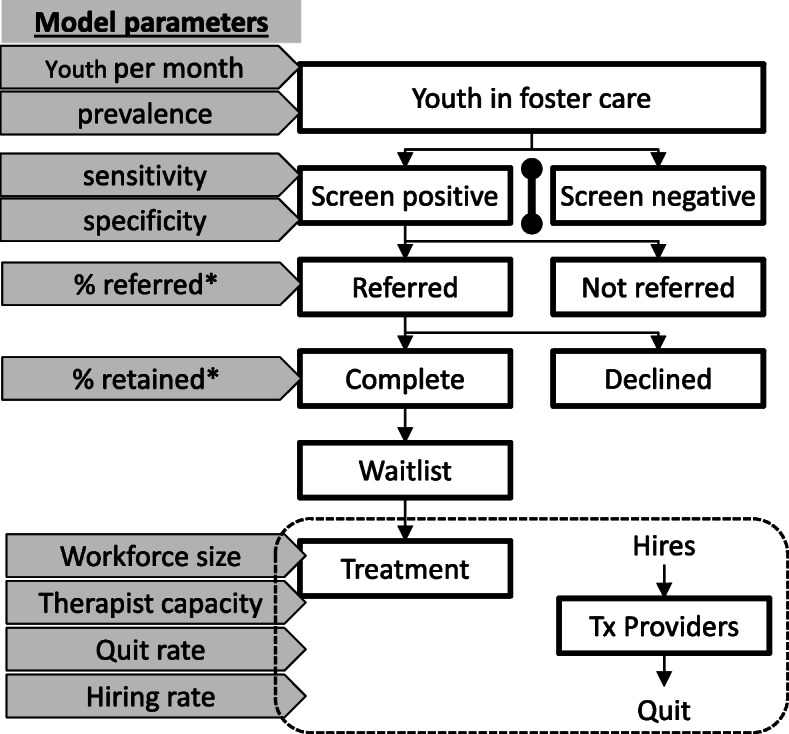
Baseline Monte-Carlo model of a screening process. Note. *separate parameters were specified for youth with and without trauma, who may differ with respect to chance of referral and retention

Specifically, the modeling team adapted a prior simulation model [12], conducted virtual experiments, and created a presentation to communicate a description of model structure and key findings. Respondents had no prior experience with simulation modeling; therefore, the presentation was designed to introduce key concepts and practical applications of the model, as well as potential insights. Although this paper is not intended to validate a simulation model, we present enough detail to demonstrate how modeling functioned in the RCSM process. The baseline model (Fig. 1) depicts discrete steps of the system of care in which screening is situated, beginning with the screen itself and then moving to the referral decision and outcome, culminating in a treatment queue. A separate model depicts the workforce available to provide that treatment.

**Fig. 2:**
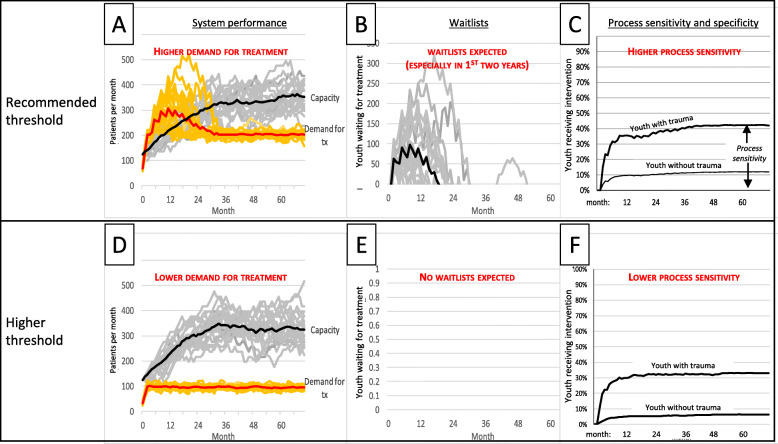
Influence of screening threshold on system capacity, demand for treatment, and waitlists. Note. **A–E** display 20 different runs of the simulation model, each of which reflects a possible trajectory that is consistent with model assumptions yet differs because of stochastic elements inherent in the process. Darkened lines represent average values. Note that intervals around system capacity, which depend on a relatively small number of treatment providers, exceed those around demand, which depend on a comparatively larger number of children receiving care through the system

Using this model, the presentation addressed topics relevant to stakeholder questions:

1. *Downstream system capacity to respond*. The baseline model was specifically designed to guide discussion about whether system treatment capacity is sufficient to meet demand resulting from screening. Lacking the time and data necessary for accurate, system-specific predictions, we focused on conceptual issues, such as which variables might govern demand for treatment after screening implementation. Therefore, the presentation included questions about the plausibility of model parameters for the probability of referral and its completion, including whether such parameters were likely to be equivalent for children with and without trauma. Notably, these questions touch on scientific debates about the utility of clinical decision-making subsequent to the use of quantitative screening tools [62–65]. In addition, recent publications highlight the role of workplace burden on provider burnout [66]. Therefore, phase 3 member-checking group interviews inquired about the extent to which waitlists might influence (i.e., feedback to) other model variables governing referral decisions, referral completion rates, and provider quit rates.
2. *Threshold or “cut-score” for referral*. To address stakeholders’ questions regarding screening thresholds, sensitivity analyses simulated tradeoffs from raising screening thresholds. Consistent with our team’s past research [20, 62], Fig. 2 depicts the influence of screening thresholds on system performance (demand for treatment and treatment capacity; Fig. 2a, d), waitlists (Fig. 2b, e), and process sensitivity and specificity (Fig. 2c, f). The top row of panels in Fig. 2 do so under the assumption that recommended screening thresholds are implemented, while the bottom row depicts results under the assumption that screening tools are scored using a higher threshold. The model demonstrates that a higher threshold may result in shorter waitlists, but fewer children receiving treatment.

(3) *Capacity to start-up and sustain screening*. Simulation revealed that initial assumptions regarding when the treatment workforce was hired resulted in a lag in increased system capacity, thus leading to a risk of waitlists in the first 2 years of the baseline model. In short, waiting for demand to increase before hiring new treatment providers could result in significant waitlists before supply catches up with demand. This issue was not anticipated by the research team and was discussed in the presentation.
(4) *Screening program reach.* In the model, a single parameter determines the proportion of the population that receives screening. The presentation also emphasized that parameters could be adapted to reflect different populations; for example, young children might display different prevalence of trauma than adolescents and accordingly be eligible for different services. Therefore, the presentation included questions about the utility of adapting the model to address program reach.
(5) *Screening tool content*. The presentation noted that different model parameters may reflect different operational definitions of trauma. For example, a screening instrument may be validated using a structured interview that offers one definition, whereas clinicians may find benefit in treating children who are “subthreshold” by formal diagnostic criteria. In this case, a “false positive” by one definition may be a “true positive” by another. Moreover, we noted that developmental and behavioral problems can be conceptualized not only as a binary diagnostic classification, but also as a continuum. Therefore, prevalence can be more than just a single number and can vary over time and place [67] and youth’s years of exposure [68, 69]. Thus, the presentation included questions not only about the plausibility of the model’s prevalence estimate, but also about the nature of the problem to be addressed and whether there is likely to be consensus among all participants in the screening process.

### RCSM Step 3: Engage stakeholders

The goal of the third step is to assess model relevance, potential for insight, and utility to inform implementation decisions. With respect to relevance, respondents reported that the model generated an accurate representation of the decisions confronted and tradeoffs considered when developing their respective screening protocols. Illustrative of this theme, one respondent stated, “Oh yeah, these are kind of typical points of conversation, questions, decision-making that we run into.” Respondents also indicated the availability of data sources required to parameterize the model within their respective administrative data systems, suggesting the feasibility of tailoring simulation models to their specific systems. Despite general agreement that model parameters were plausible, respondents noted that local data could facilitate system-specific estimates.

With respect to insight, respondents articulated multiple ways that the simulation model influenced their mental models of screening implementation. First, the model reinforced participants’ inclination to attend to *overall process sensitivity* rather than the sensitivity of the screener alone. The model also promoted consideration of alternative intervention strategies, such as care coordination or “warm hand-offs,” to improve overall process sensitivity. As one respondent articulated:

The challenge we see is from referred to completion because that's where you run into the wait times, the different providers, the lack of capacity, or the intervention of someone with a disagreement or that thinks because a child is stable in care, they don't need mental health services. Things like that. So that's an active area that we'll actually be exploring is how to create that automated pathway to make sure that the referral results in a warm care coordination handoff to ongoing care. -FG 1

Second, the model provided insight into potential modifications to the screening process where service capacity was not adequate. Respondents routinely reflected on whether thresholds should be adjusted depending on the downstream capacity of delivery systems, as illustrated in the following quote:

– it does beg the question, should you have differing screening criteria based on the area? But that is mostly driven by capacity, to be totally honest. -FG 4

Moreover, model results suggest the time required to hire treatment providers will result in a time lag for treatment supply. The implications of this assumption for waitlists only became clear through the simulation process. As noted by one participant:

I mean this is the kind of thing that you in hindsight wish that the people with the good intentions had had in front of them before they actually put the legislation forward or were able to account for the consequences that would inevitably come with major policy changes. Rather than just saying well, this is the right thing to do so, you know, we're just going to do it and deal with the consequences, actually having a … more technical conversation about the expected implications. -FG 1

In turn, questions were raised that were not anticipated by our research team, such as the possibility of adapting the model to compare performance across county-level systems rather than only optimizing performance in a single system. In addition, respondents questioned the model structure by noting that referral decisions were often clinically informed rather than determined solely by screening instruments—an observation that was consistent with the research team’s past research but was not reflected in the simplified model [65]. These insights would be important to addressing stakeholder needs in successive iterations of RCSM.

In regard to RCSM’s utility as an implementation strategy, respondents indicated that the model structure would facilitate dialogue about implementation, potentially altering “mental models” of key stakeholders, including system partners and researchers. Illustrative of this, one respondent articulated the model’s utility for building new understandings among system partners:

I wouldn’t say it’s obvious, like if you look across the different systems that would interface with this, so again, saying that if this is mental health and you have wait lists for kids that do qualify that's hugely problematic but at least we know they have a need … I think it makes sense in my mind, *but I don’t think that our partners think about it in this way with the addition of thinking about how it impacts other system partners and other dynamics of the system of care.* -FG 1

Policymakers also articulated how RCSM could facilitate communication with researchers:

I do know that [screening tool developer], who developed the tool, feels very strongly that it’s a good indicator of what needs to happen, and they’d like to see our thresholds much lower than what they are for the kind of intervention. So, I think, if anything, *it might help the developer in our department feel better about what we've set as potential thresholds*. Whether or not they would welcome that, I don’t know. -FG 2

These statements suggest how RCSM could be used to promote dialogue and achieve cultural exchange both prior to and during implementation efforts.

## Discussion

Results reaffirm the use of simulation modeling as an implementation strategy. Asking stakeholders about implementation decisions before developing the simulation model (i.e., the design phase) resulted in a model decision-makers found relevant to a set of necessary decision points. Decision-makers reported gaining insight into how system variables can impact the success of universal screening protocol and how investments in “hand-offs” and treatment system capacity may complement screening by improving overall system performance. In turn, researchers gained further insight into the needs of decision-makers, such as the possibility of county-level models to consider targeting resources within a given state. Both groups reported insight into the importance of timing hiring to anticipate increases in demand.

The relative simplicity of the model helped to facilitate this insight. As Hovmand notes [44], “Simply helping groups recognize that there is a system, the components that constitute the system, or how the components might be related through feedback can readily solve some problems,” (p. 49). In our case study, participants were able to challenge structural assumptions in the model, such as the extent to which referrals were determined by screening (as opposed to attrition at each stage of the screening process) and the possible influence of waitlists in influencing supply and demand of treatment through feedback loops. At a deeper level, the model facilitated dialogue regarding differences in the meaning of “trauma”—a concept central to determining eligibility and tracking progress.

Consistent with cultural exchange theory, the case study demonstrated the importance of dialogue—both among implementers and with researchers. The question of screening thresholds is a case-in-point. Whereas researchers often use receiver operating characteristics (ROC) curves to balance sensitivity and specificity, one respondent received affirmation for the view that thresholds are “mostly driven by capacity.” This difference in perspective mirrors a debate in the research literature [63, 65], and respondents reported that the model could be useful for facilitating conversations with researchers who hold different views.

We note several limitations. While we ground RCSM in contemporary epistemology, by no means have we conducted a comprehensive review of this subject. Moreover, by emphasizing the rapid application of simple models, RCSM merely scratches the surface of the potential inherent in more complex simulation models, such as recent advances that integrate policy-relevant decision models with system dynamics to directly address rapidly changing contexts [70]. We invite comment and critique from philosophers and expert modelers, particularly those familiar with previous efforts to disseminate system dynamics concepts [58, 71, 72].

In addition, we make no claim that modeling and dialogue guarantee insight; at best, they create fertile soil for insight to germinate. Indeed, the single round of RCSM in our case study offer proof-of-principle regarding the inquiry stage of dialogue, but additional research is clearly needed. With regard to process, more advanced facilitation techniques may be needed to ensure productive critical discussion and deliberation, where the goal is to reveal truth and determine the best course of action while avoiding simple debate, where the goal is often to win regardless of the truth underlying one’s position [42]. In addition, guidance is needed to inform decisions regarding the need for additional iterations of RCSM, for example by articulating potential benefits (e.g., by engaging additional stakeholder or avoiding premature closure) and costs (e.g., taxing available capacity, or exceeding “policy windows”). Ultimately, the key questions are whether engaging (and continuing to engage) in RCSM meaningfully improves decision-makers’ use of available evidence, and in turn whether such use improves outcomes valued by key stakeholders.

## Conclusions

With limitations in mind, results suggest RCSM’s potential to extend use of simulation modeling both as an analytic strategy for evidence synthesis and as an implementation strategy to promote dialogue regarding underlying assumptions, shared reasoning to the best explanation for available evidence, and evidence-informed decision-making regarding optimal courses of action.

## Data Availability

Due to their qualitative nature, data generated and analyzed during the current study are not publicly available.
